# Implantation of regenerative complexes in traumatic brain injury canine models enhances the reconstruction of neural networks and motor function recovery

**DOI:** 10.7150/thno.50540

**Published:** 2021-01-01

**Authors:** Jipeng Jiang, Chen Dai, Xiaoyin Liu, Lujia Dai, Ruixin Li, Ke Ma, Huiyou Xu, Fei Zhao, Zhiwen Zhang, Tao He, Xuegang Niu, Xuyi Chen, Sai Zhang

**Affiliations:** 1 Tianjin Key Laboratory of Neurotrauma Repair, Institute of Traumatic Brain Injury and Neuroscience, Center for Neurology and Neurosurgery of Characteristic Medical Center of Chinese People's Armed Police Force (PAP), Chenglin Road No.220, Tianjin 300162, China.; 2 Postgraduate school, Medical school of Chinese People's Liberation Army (PLA), General Hospital of PLA, Fuxing Road No. 28, Beijing 100853, China.; 3 Department of Neurosurgery, West China Hospital, West China Medical School, Sichuan University, Chengdu, Sichuan 610041, China; 4 Department of Neurology, the Armed Police Corps Hospital of Zhejiang Province, Hangzhou 310000, China.; 5 Tianjin Key Laboratory of Oral and Maxillofacial Function Reconstruction, Tianjin Stomatological Hospital, Tianjin 300041, China.; 6 SUSTech-UTokyo Joint Research Center on Super Smart City, Southern University of Science and Technology, Center for Spatial Information Science, The University of Tokyo, Tokyo 277-8568, Japan.; 7 Department of pathology, Characteristic Medical Center of Chinese People's Armed Police Force (PAP), Chenglin Road No.220, Tianjin 300162, China.; 8 Department of Neurosurgery of Tianjin Fourth Central Hospital, Zhongshan Road No.1, Tianjin 300143, China.

**Keywords:** Hemiplegic limb, Collagen, Silk fibroin, Mesenchymal stem cell, Traumatic brain injury

## Abstract

**Rationale:** The combination of medical and tissue engineering in neural regeneration studies is a promising field. Collagen, silk fibroin and seed cells are suitable options and have been widely used in the repair of spinal cord injury. In this study, we aimed to determine whether the implantation of a complex fabricated with collagen/silk fibroin (SF) and the human umbilical cord mesenchymal stem cells (hUCMSCs) can promote cerebral cortex repair and motor functional recovery in a canine model of traumatic brain injury (TBI).

**Methods:** A porous scaffold was fabricated with cross-linked collagen and SF. Its physical properties and degeneration rate were measured. The scaffolds were co-cultured with hUCMSCs after which an implantable complex was formed. After complex implantation to a canine model of TBI, the motor evoked potential (MEP) and magnetic resonance imaging (MRI) were used to evaluate the integrity of the cerebral cortex. The neurologic score, motion capture, surface electromyography (sEMG), and vertical ground reaction force (vGRF) were measured in the analysis of motor functions. In vitro analysis of inflammation levels was performed by Elisa while immunohistochemistry was used in track the fate of hUCMSCs. In situ hybridization, transmission electron microscope, and immunofluorescence were used to assess neural and vascular regeneration.

**Results:** Favorable physical properties, suitable degradation rate, and biocompatibility were observed in the collagen/SF scaffolds. The group with complex implantation exhibited the best cerebral cortex integrity and motor functions. The implantation also led to the regeneration of more blood vessels and nerve fibers, less glial fibers, and inflammatory factors.

**Conclusion:** Implantation of this complex enhanced therapy in traumatic brain injury (TBI) through structural repair and functional recovery. These effects exhibit the translational prospects for the clinical application of this complex.

## Introduction

There are rising incidences of traumatic brain injuries (TBI) in global public health. Globally, at least 50 million new cases of mild TBI and 2 million new cases of moderate/severe TBI (sTBI) occur annually 1. Patients with TBI, especially those with sTBI exhibit dismal prognostic outcomes. This condition has been associated with physical disabilities that render thousands of young people jobless of unable to self-feed 2.

Traditional rescue surgical procedures, conventional medicines, and functional exercises are important therapeutic options. However, they have limited efficacies. Multiple complications such as ischemia and hypoxia in the traumatic area, poor nutritional status, deficiency in neurotrophic factors, excessive trauma, severe glial hyperplasia, and inflammatory reactions constitute physical and chemical barriers in the microenvironment 3, 4, These complications lead to poor prognosis by inhibiting the regeneration and extension of axons. Advances in stem cell-based tissue engineering technology has provided a promising therapeutic option for TBI. Functional tissues or organoids are being developed through a combination of stem cells, neurotrophic factors and biomaterials to allow for more axonal growth and synaptic connections 5, 6. Mesenchymal stem cells (MSCs) are effective in immune modulation, enhancing angiogenesis, neural protection, anti-inflammation, suppressing cavity formation, and impeding glial scars 7. However, the fate of mesenchymal stem cells in the harsh TBI microenvironment has not been established. Therefore, the provision of a stable microenvironment for these cells is of great significance 8. Porous biomaterials improve the survival of transplanted stem cells 9. Collagen and silk fibroin (SF) are natural materials with favorable biocompatibility and biodegradability properties 10. Previous studies have established the safety and efficacy of collagen and SF in peripheral nerve and spinal cord regeneration 11, 12. To improve the carrying efficiency of stem cells and to provide a bridge for creeping regenerated axons and nerve fibers during the repair period, we fabricated a small, porous scaffold made of collagen and SF for the colonization and proliferation of MSCs.

Various scaffolds have been shown to be effective in the field of neural trauma repair. However, their clinical applications have not been confirmed 13. TBI has been associated with the loss of nerve tissues. Compared to small animals, TBI leads to the development of irregular shaped lesions in humans due to different damage factors and mechanisms 14. Most TBI studies have been performed in rodent models 15. Because of the tremendous differences in brain structure, these TBI model rodents exhibit relatively low physiological and pathological similarities with clinical features of human TBI 16. In addition, the evaluation methods for effective TBI repair in many studies are singular and unobjective 17. From both anatomical and functional perspectives, a limited number of studies have objectively revealed locomotory improvements of hemiplegic limbs after TBI repair 16.

We constructed a biological complex made up of a scaffold and MSCs to simulate the occurrence and development of TBI in canines. Objective and accurate techniques such as motion capture, RNA in situ hybridization (ISH), and genome sequencing were used to evaluate the repair effects of the complex on TBI.

## Materials and methods

### Experimental animals and ethical statement

The animals involved in this study were male adult (1 year old) beagles (Fang Yuanyuan Experimental Animal Center, Beijing, China. License number: SK 2014-0012, batch number: NO. 11804900010039). Their average weight was 10 kg (range from 9-11.5 kg). They were maintained in an animal house with drinking water and food being provided ad libitum. Ethical approval for this study was obtained from the Institutional Animal Care and Use Committee of the Chinese People's Armed Police Force (PAP) Medical Center (approval number is PJHEC-2017-01 (AF)).

### Preparation of collagen/SF scaffold

Fresh bovine tendons were washed and the adventitia and adipose tissues removed. The tendons were crushed and soaked in 0.05 Mtris buffer (Yacoo Science Co., Ltd., Suzhou, China) solution for 24 h to remove impurities. After centrifugation at 2000 rpm, the sediments were collected. Acetic acid solution containing pepsin was added to the sediment and centrifuged to obtain the supernatant. NaCl solution was added to the supernatant and the sediments obtained by centrifugation after salting out. The collagen gel was then obtained by dialysis. Silk (Kaidi Silk Co., Ltd., Jiangsu, China) was prepared and soaked in 0.5% Na_2_CO_3_ (Solarbio Science & Technology Co., Ltd., Beijing, China) solution for 30 min and dehydrated, CaCl_2_·CH_3_CH_2_OH·H_2_O (Solarbio Science & Technology Co., Ltd., Beijing, China) solution was added and the temperature maintained at 70 ℃, After filtration, dialysis, and concentration, the SF solution was obtained. Gelatin (2 g) (Tianjin Kermel Chemical Co., Ltd. China) was dissolved in 50 mL of 3% SF solution (60 ℃, 30 min), after cooling, 25 g collagen gel (3%) was added to the solution and completely stirred. The prepared mixed gel contained collagen, SF, and gelatin. The mixed gel was then retained in a syringe and extruded evenly into a coagulation bath (NaOH-ethanol solution, 4 ℃) at a speed of 1 mm/s. The solidified gel strips were removed and freeze-dried. Porous collagen/SF scaffolds were prepared by immersing the freeze-dried gel strips in absolute ethanol and purified water (30 ℃) successively for 24 h after dissolution and precipitation of gelatin. Scaffolds were cut into particles (2 mm × 2 mm) and sterilized with Co 60 radiation.

### Physical property detection and degradation experiment of collagen/SF scaffold

The general structures of the scaffolds with or without water were observed under the operating microscope. Scaffolds were then fixed with 2% glutaraldehyde and 1% osmium tetroxide (Sigma, St. Louis, MO, USA), dehydrated with gradient acetone and rapidly frozen by liquid nitrogen and critical point dried, the microscopic morphologies of scaffolds were observed by a scanning electron microscope (SEM; QUANTA200, FEI, Eindhoven, the Netherlands).

The scaffolds (10 mg) were heated at a rate of 10 K/min in an argon atmosphere. The samples were gradually heated from 25 ℃ to 400 ℃. The phase transition temperature of the scaffold during heating was analyzed by the synchronous differential thermal analyzer (STA409PC/PG, Germany).

The scaffolds were scanned by Bruker X-ray Diffraction (AXS D8 Advance, Germany) for crystallization analysis. Cu (Ka=0.15406 nm) was selected as the target source material. The scanning current was 30 mA, the voltage was 40 Kv, the scanning angle was 5°-90°, the step width was 0.02°, and the scanning speed was 5°/min.

Scaffolds were ground into powder, the sample was prepared by KBr pellet and its properties determined by Nicolet 870 infrared spectroscopy (Nicolet, USA). The scanning range was 600 cm^-1^-4000 cm^-1^, the resolution was 4 cm^-1^, and the scanning speed was set at 1 cm/s. Twenty times of scans were performed.

Scaffolds were immersed in a PBS (0.01 mol/L, pH=7.4) solution for 24 h. The compressive mechanical properties (n = 5) were determined using the Instron 5865 mechanical testing machine (Instron, USA). The preload was set as 0.1 N, the sinusoidal waveform was 5 Hz, and the maximum compressive strain was 50%. The compressive stress-strain curve was drawn and the elastic modulus calculated.

Experiments to determine the degradation rates of the scaffolds were performed on 8 canines. These experiments were performed under general anesthesia. Briefly, the head was shaved and sterilized. An incision (1 cm in length) was made on the left scalp. The skin was separated from the subcutaneous tissue. A single collagen/SF scaffold was fixed to the subcutaneous area. The skin and subcutaneous tissue were sutured and a mark made on the scalp. Anesthesia, preoperative preparation, and postoperative care were performed as described previously 16.

At each time point (1, 2, 4, and 8 weeks) after the operation, two canines were randomly chosen and anesthetized in order to remove the implanted scaffolds. The scaffold material was cleaned with distilled water and dried to a constant weight in a vacuum. The degradation rates of the scaffolds were then calculated by the formula: Degradation rate=(W1-W2)/W1×100%. Whereby, W1 is the quality of scaffolds before degradation and W2 is the quality of residual scaffolds after degradation.

### Extraction and identification of human umbilical cord mesenchymal stem cells

The umbilical cords of healthy full-term newborns were washed (Approved by the ethics committee), cut and digested. hUCMSCs were separated and inoculated in serum-free conditioned medium (Sciencell, USA) at a density of 1×10^6^/mL and cultured in a 37 ℃, 5% CO_2_ incubator for two days. The suspended cells were removed and passaged at an inoculation density of 5×10^3^/cm^2^. hUCMSCs were cultured to the third generation. Specific surface antigens were detected by flow cytometry. After trypsinized digestion, the cells were washed three times with PBS solution and a suspension (density of 1×10^6^/mL) made. The cells were packed with CD90-FITC, CD105-PerCP-Cy5.5, and CD73 APC (Abcam, Cambridge, UK) in 0.1 mL. The positive rate of antigen was detected by flow cytometry after incubation at 4 ℃ for 30 min.

### Fabrication of implantable complexes and the biocompatibility and toxicity tests of collagen/SF scaffolds

A hundred μL hUCMSCs were inoculated into the scaffold at a density of 10^7^/mL. After 7 d of culture, cell morphology and proliferation in the pores of scaffolds were observed under an inverted phase-contrast microscope. The co-culture complexes were fixed with 2% glutaraldehyde and 1% osmium tetroxide, dehydrated with gradient acetone, and rapidly frozen by liquid nitrogen. The critical points were then dried and microscopic morphologies of the complexes observed by SEM. The complexes were immersed in 4% polyformaldehyde solution (pH=7.4, Solarbio Science & Technology Co., Ltd., Beijing, China). After 24 h of immobilization, they were made into paraffin-embedded materials and cut into slices (5 µm) for HE staining. The cell growth of hUCMSCs in scaffold were observed under a microscope. The complexes were also cut into frozen slices (10 µm) and incubated overnight (4 ℃) with anti-CD90 (mouse anti-human, 1:200, Bioss, Beijing, China) and anti-CD105 (Rabbit anti-human, 1:200, Bioss, Beijing, China). After overnight incubation, the slices were again incubated with a second antibody for 1 h (37 ℃). The hUCMSCs markers for CD90 and CD105 on scaffolds were observed using an inverted fluorescence microscope (DMI4000B, Leica, Germany).

A hundred μL third-generation hUCMSCs were inoculated on pre-wetted scaffolds at a density of 10^4^/mL in 96-well plates. Five duplicate wells were set in each group. The hUCMSCs adherent culture was set as the control group. The serum-free culture medium was replaced after every two days. For every time that 300 uL new culture medium was added to the wells, 30 uL of CCK-8 (Abcam, Cambridge, UK) solution was also added under dark conditions at 1 d, 3 d, 5 d and 7 d respectively. Incubation was done at 37 ℃ in 5% CO_2_ for 2 h. The OD values were measured at a wavelength of 450 nm and growth curve plotted.

### Hemiplegic model development of traumatic brain injury in canines and implantation of complexes

A total of 24 canines were used in this study. The animals were randomly distributed into four groups: The TBI group (n = 6), the stem cell (SC) group (n = 6), the Collagen/SF scaffold (CS) group (n = 6) and the combination group (Collagen/SF scaffold combined with stem cell, CB, n = 6) (**Figure 2J**) (In the present study, we didn't set Sham group since we have proved that small area craniotomy and dura incision have no adverse effects on canines, there is no significant difference between the sham operated group and control group on neurological function) (**Figure S1 & Figure S2**) 16.

The TBI model was established as previously described 16. After complete hemostasis, a suspension (1 mL) of stem cells (1×10^7^ cells) or collagen/SF scaffolds (with or without stem cells) were implanted into the trauma foci using a syringe (**Figure 2K-M**). The lesion was sewed using an artificial dura mater by the relaxation suture method to make sure that the transplants would not leak out. All the above animal procedures were performed under general anesthesia.

### Detection of motor evoked potential

Postoperative detection of electrophysiology was initiated at 1, 3, and 6 months after implantation. Time associated changes in the amplitude and latency of MEP and differences among groups were determined. This determination was achieved by applying transcranial constant pressure stimulations to the scalp projection area of the motor cortex and detecting the latency and amplitude of MEP in the extremities. Briefly, two needles were fixed into the cranial muscles and used as the stimulating electrodes, while two more needles were inserted into each of the muscles of extremities (Forelimb: the extensor carpi radialis, Hindlimb: the biceps femoris (BF)) and used as the detecting electrodes. Additionally, an electrode was fixed on the tail as the grounding wire. Four levels (90 V, 100 V, 110 V, 120 V) of stimulating voltage (Pulse-width: 0.5 ms, stimulus frequency: 500 Hz) were selected during each detection. The results were displayed and recorded on the evoked potential (EP) instrument Viking Quest system (Thermo Nicolet Corporation, USA).

### Diffusion tensor imaging and magnetic resonance spectroscopy imaging

Magnetic resonance imaging (MRI) scan and diffusion tensor imaging (DTI) construction were applied in the identification of the integrity of cerebral cortex and corticospinal tract under the Siemens MAGNETOM Verso 3.0T MRI system. This system is composed of three phase including, the T1 phase (Field of view (Fov) read: 200 mm × 200 mm, Fov phase: 100%, slice thickness: 1 mm, repetition time (TR): 1900 ms, echo time (ET):2.64 ms), the T2 phase (Fov read: 206 mm × 206 mm, Fov phase: 100%, slice thickness: 1 mm, repetition time (TR): 3200 ms, echo time (ET): 411 ms) and the DTI construction phase (Fov read: 206 mm × 206 mm, Fov phase: 100%, slice thickness: 4 mm, repetition time (TR): 6400 ms, echo time (ET): 95 ms).

### Limb behavior assessment and motor function evaluation

Neurological function evaluation involved two aspects. After 1 d, 1 week, 2 weeks, 4 weeks, 8 weeks, 12 weeks, 16 weeks, 20 weeks and 24 weeks post-surgery, a series of scoring scales including modified Glasgow Coma Scale (mGCS) 18, Purdy scale 19 and NDS scale 20 were used to comprehensively evaluate the level of neurological functions including consciousness, reflex, behavioral characteristics, and motor functions among others. The score ranges for mGCS were 3-18 (3: brain damage, 18: represent health), Purdy score were from 2 to 11 (2: health, 11: coma or death) while the NDS score ranges were 0-500 (0: health, 500: brain damage). Scoring was done by two laboratory technicians who were blinded from this study. The final score was defined as the average score from the readings of the two technicians.

### Gait analysis of limbs with a motion capture system

The motion capture system (Oxford Metrics Limited, UK) was used in the precise analysis of gait characteristics **(Figure 5E)**. Briefly, a treadmill and six high-speed cameras were arranged. Four marker balls with fluorescent layers were then pasted on the major joints of each side of the canine's hindlimbs. The markers were labeled as left/right joint A, B, C and D from the upper joint to the lower joint. An endpoint was set up and used as the reference point for height change measurements after every pace. In the motion capture system, motion amplitude, real-time joint trajectory, angle variations, and height changes were reflected and recorded. Before motion capture, canines must be trained to adapt to the rhythm of the treadmill and walk with hindlimbs when the forelimbs were fixed. Before TBI modeling, each canine walked a distance of 0.75-1km (mileage displayed on the treadmill) every day. After TBI, the hemiplegic canines walked for about 0.1-0.2 kms every day. After months of training, all the 24 canines in the four groups can walk on the treadmill at the speed of 1.5 km/h.

### Surface electromyography and muscle strength testing

DTS EMG sensor system (Noraxon, USA) and AMTI force platform (Advanced Mechanical Technology Inc, USA) were used to detect the real-time surface electromyography (sEMG) changes of major muscle groups and vertical ground reaction force (vGRF) **(Figure 5E)**. In the sEMG signal acquisition process, two electrodes and a sensor were tightly attached to the bilateral skin of the BF muscle. Sampling frequency was set as 1500 Hz. In addition, the treadmill and canine were placed on a force platform with the force value zeroed. The canines' forelimbs were fixed on the treadmill and the vGRF of the hindlimbs synchronously measured. These detections were recorded using the Vicon Nexus software.

### Gross and staining observation of cerebral cortex repair

Six months after the operation, the animals were sacrificed after general anesthesia. The brains were cut into 2 mm-thick slices across the lesion and the gross morphology of the lesions in the cerebral cortex observed. Masson staining was done to observe the proliferation of glial fibers. Paraffin sections (5 µm thick) were prepared. The sections were then dewaxed with xylene, dehydrated with gradient alcohol and washed with distilled water. After hematoxylin staining, the nucleus was stained with ponceau S (Solarbio Science & Technology Co., Ltd., Beijing, China) and rinsed with distilled water. They were then treated with phosphomolybdic acid. The aniline blue dye and glacial acetic acid (Solarbio Science & Technology Co., Ltd., Beijing, China) were used for staining the sections before observation under a microscope. Then Luxol Fast Blue (LFB) staining was performed as described. First, the paraffin sections (5 µm) were dewaxed and immersed into the LFB dye (Solarbio Science & Technology Co., Ltd., Beijing, China) solution (1%) and dyed for 2-4 h at 60 ℃. The sections were then soaked in ethanol and lithium carbonate (Solarbio Science & Technology Co., Ltd., Beijing, China) solution and taken out immediately. Until the background getting light and the color of blue appearing completely, sections were lastly dehydrated, sealed, and observed under a microscope. Silver staining was also performed in this study. After dewaxing, the paraffin sections (5 µm) were washed with distilled water and incubated in silver nitrate (Sigma, St. Louis, MO, USA) solution (2%, 37 ℃) in the dark for 30 min. The slices were then washed with distilled water and formaldehyde (Sigma, St. Louis, MO, USA) solution (10%) for 5 min and placed in a wet box. The slices were again washed until they turned to dark brown color and subsequently transferred into sodium sulfate (Sigma, St. Louis, MO, USA) solution (5%). After washing, the slices were sealed and observed under a microscope.

### mRNA in situ hybridization and immunofluorescence staining

Critical markers for angiogenesis (vWF: von Willebrand factor), myelination (MBP: myelin basic protein), dendrites and axonal regeneration (MAP-2: microtubule-associated protein-2, NEFM: neurofilament M) and synapse formation (Syn: synapsin) were detected. *In situ* hybridization was performed using the multichannel fluorescent kit (323100, Advanced Cell Diagnostics, USA) according to the manufacturer's instructions. Probes against canine vWF (543711), MBP (300031-C2), NEFM (320269-C2), MAP-2 (555831-C2), and Syn (479111-C2) were obtained from Advanced Cell Diagnostics, Inc. Immunofluorescence staining was used to detect the expression of corresponding proteins. The paraffin slices were dewaxed and EDTA buffer (Abcam, Cambridge, UK) solution used to repair the antigen. Slices were then treated with BSA (Abcam, Cambridge, UK) and incubated for 30 min. Primary antibodies vWF (MBP-NEFM, Syn, MAP-2) (vWF: Rabbit anti, 1:1000, Abcam, Cambridge, UK; MBP: Rat anti, 1:1000, Abcam, Cambridge, UK; NEFM: Rabbit anti, 1:1000, Proteintech, USA; Syn: Rabbit anti, 1:1000, Bioss, Beijing, China; MAP-2: Chicken anti, 1:1000, Abcam, Cambridge, UK) were added to the slices and incubated in a wet box at 4 ℃ overnight. The primary antibodies were then removed from the slices and secondary antibodies added. They were incubated at room temperature for 50 min. DAPI (Sigma, St. Louis, MO, USA) staining was done and after 10 min of incubation at room temperature, the slices were sealed and observed under a fluorescence microscope (DMI4000B, Leica, Germany).

### Transmission electron microscope scanning and cell fate study

Six months after the surgical procedures, the filling tissue of cerebral cortex lesions were cut into small pieces and fixed with glutaraldehyde (2.5%) at 4 ℃ overnight. They were then fixed with 1% osmium acid for 1.5 h. Dehydration with gradient ethanol and overnight polymerization in epoxy encapsulated medium (Sigma, St. Louis, MO, USA) was done. The tissues were settled for 10 h at 60℃, ultra-thin slices prepared by Ultramicrotome (Leica EM UC7, EMS, USA) and stained with uranium acetate and lead citrate (EMS, USA). Myelin sheath and newborn synapses were observed under a transmission electron microscope (TEM, 80 KV, Zeiss, Germany). The fate of hUCMSCs was also determined. The paraffin slices were dewaxed and the EDTA buffer solution used to repair the antigen. The slices were then treated with BSA, incubated for 30 min, and CD90 (mouse anti-human, 1:200, Bioss, Beijing, China) and CD105 (Rabbit anti-human, 1:200, Bioss, Beijing, China) mixtures added. Secondary antibodies and DAPI were successively used, the slices were sealed, CD90 and CD105 co-expressed cells were observed under a fluorescence microscope. In order to track the cell fate of the hUCMSCs in SC and CB group, we arranged immunohistochemistry of Doublecortin (DCX, Rabbit anti-human, 1:1000, Abcam, Cambridge, UK) and NeuN (Rabbit anti-human, 1:1000, Abcam, Cambridge, UK) staining.

### Detection of plasma inflammatory factors

Peripheral venous blood plasma was collected within the first week, and after 6 months of the experiment. The levels of TNF-a, IL-6, and IL-10 were measured using the Enzyme-linked Immunosorbent Assay (Elisa) (Wuhan servicebio technology Co., Ltd, China) according to the manufacturers' instructions.

### Statistical analysis

Statistical analyses were performed using SPSS 22.0 (IBM). All data were presented as the mean ± standard error. Statistical differences were compared using one-way ANOVA. Statistically significant differences in parameters were determined as ^**^p < 0.01, ^*^p < 0.05, ^##^*p* < 0.01, ^#^*p* < 0.05, ^++^*p* < 0.01, ^+^*p* < 0.05, ^ΔΔ^*p* < 0.01, ^Δ^*p* < 0.05.

## Results

### Porous collagen/SF scaffolds exhibited good physical properties and suitable degradation rates

**Figure 1:**
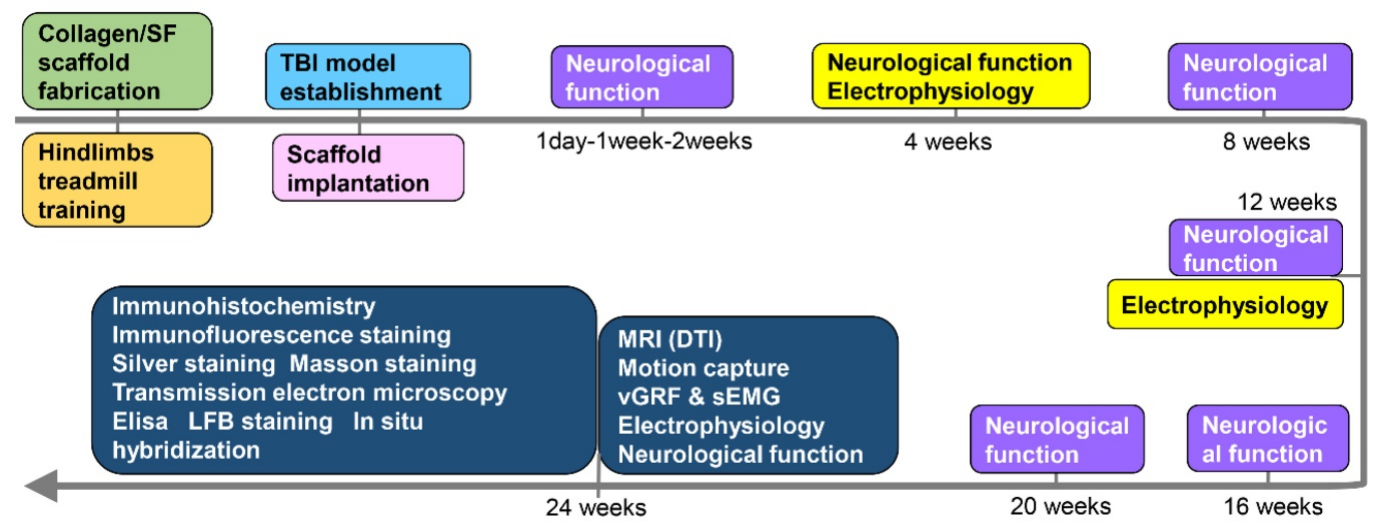
Timeline of the experiment.

**Figure 2:**
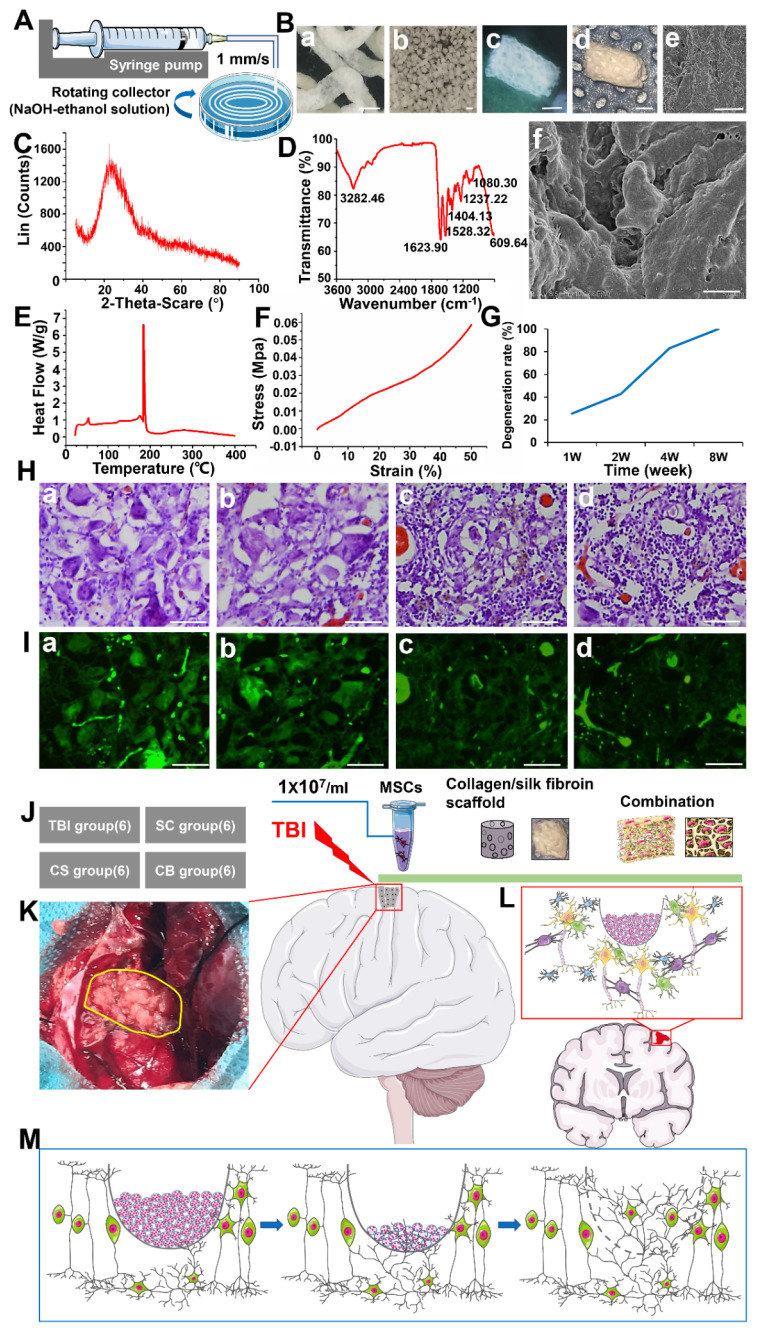
Physical property test of collagen/SF scaffolds along with the schematic profile of complexes implantation and TBI repair. (A) The fabrication process of collagen/SF scaffolds (B) General view and microstructure of collagen/SF scaffolds. Scale bars: 2 mm (a-b), 1mm (c-d) 20 µm (e), 2 µm (f). (C) X-ray diffraction of collagen/SF scaffolds. (D) Infrared spectrum detection of collagen/SF scaffolds. (E) Evaluation of collagen fibroin scaffold by differential scanning calorimetry. (F) Compressive stress detection of collagen/SF scaffolds. (G) The degradation rate of collagen/SF scaffolds (H-I) Morphological changes of collagen/SF scaffolds at the time of 1 week, 2 weeks, 4 weeks and 8 weeks under HE staining and immunofluorescence. Scale bars: 100 µm. (J) Experimental grouping and corresponding interventions. (K-M) The schematic profile of complexes implantation and TBI repair.

According to the timeline of the experimental study **(Figure 1)**, collagen/SF scaffold was firstly fabricated. The surface and interior of the scaffold were porous as observed by an operation microscope and SEM (**Figure 2A-B**). In X-ray diffraction analysis, the diffraction peak appeared at 23.72°, and the crystallinity of the scaffold changed with corresponding amount of added SF added. The collagen/SF scaffolds exhibited an ideal crystallinity, that was conducive for their stability and regulation of degradation rate (**Figure 2C**). In the infrared spectroscopy of the scaffold, the peak value at 3282.46 cm^-1^ was attributed to the stretching vibrations of C-H, the peak value at 1623.90 cm^-1^ was attributed to the stretching of C=O of the carboxyl group (COOH), the peak value at 1528.32 cm^-1^ was attributed to the stretching vibrations of NH2 double bonds. While the peak value at 1000-1475 cm^-1^ was attributed to the bending vibrations of N-H and C-N stretching. Infrared spectroscopy showed that the collagen/SF scaffolds had fat-soluble and water-soluble chemical bonds that were beneficial in cell adhesion and growth (**Figure 2D**). Collagen/SF scaffolds exhibited morphological changes and peak endothermic values during the temperature-induced endothermic process. The peak value can be used as an indicator of thermal denaturation resistance and protein stability. The endothermic peak of the scaffolds appeared between 183 ℃ and 184 ℃, at which the scaffolds decomposed. The results showed that the scaffold had a high thermal stability and was suitable for *in vivo* transplantation (**Figure 2E**). The scaffolds exhibited good ductility and plasticity. From the compressive stress-strain curve, the elastic modulus of the scaffold is calculated to be 0.07 ± 0.02 MPa (**Figure 2F**).

After transplantation, a small number of cells infiltrated into the scaffold. At 1 week post transplantation, their morphology remained intact while at 2 weeks, the structure of the scaffold became loose, with more infiltrating cells. After 4 weeks, the original morphology disappeared, and the internal structure was greatly altered with many infiltrated cells. After 8 weeks, the scaffold was completely degraded. The degradation process showed a trend of slowing down first, then increasing, and finally slowing down. The degradation rate was the fastest in the 2^nd^ to 4^th^ week post-transplantation (**Figure 2G-I**).

### Collagen/SF scaffolds exhibited good biocompatibility with human umbilical cord mesenchymal stem cells

**Figure 3:**
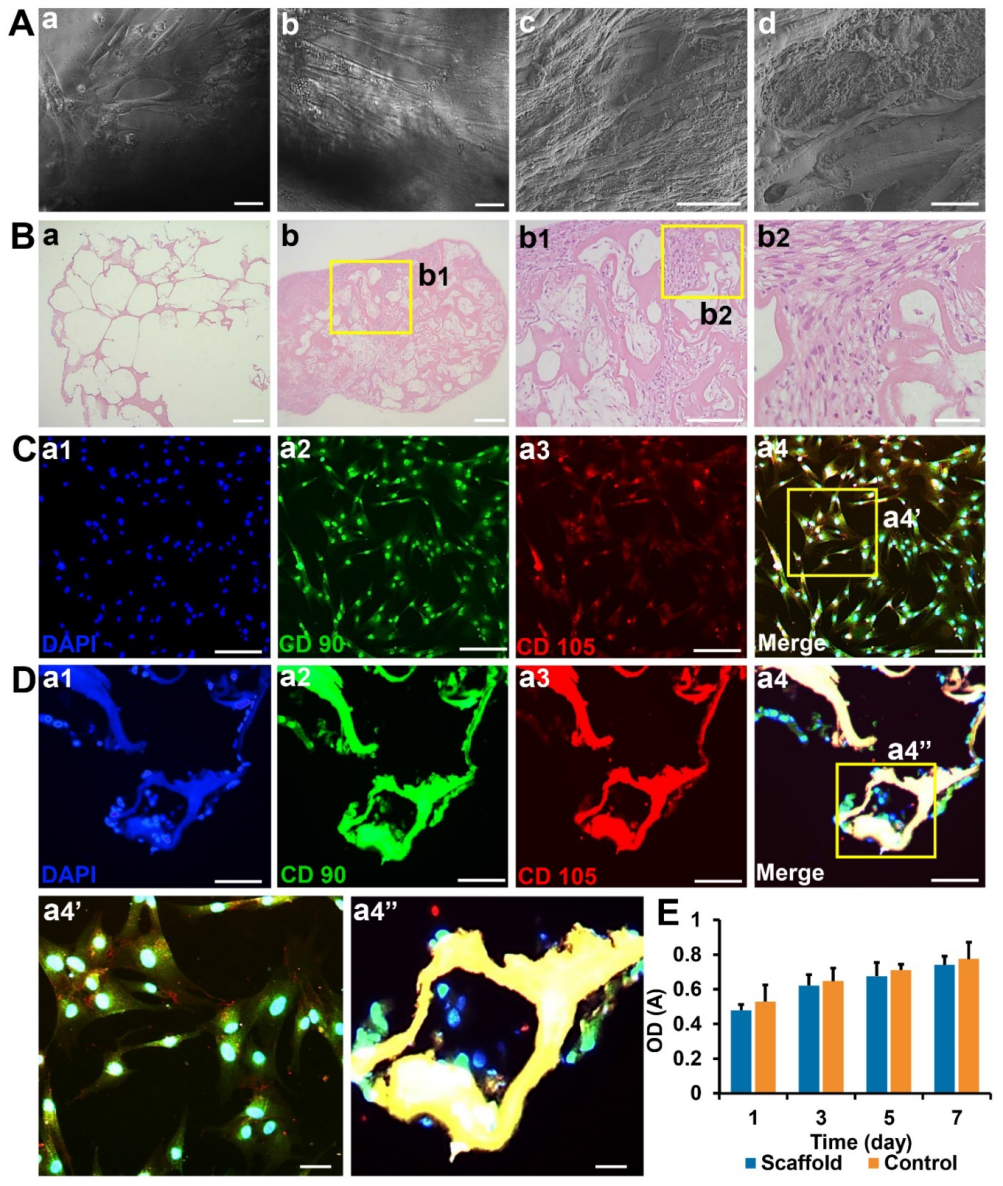
Biocompatibility and toxicity test of collagen SF scaffold (A) Observation of hUCMSCs cultured on the surface and inside of collagen/SF scaffolds under inverted phase contrast microscopy and SEM. Scale bars: 100 µm (a-c), 20 µm (d). (B) HE staining of blank scaffolds and hUCMSCs co-cultured with collagen/SF scaffolds. Scale bars: 2.5 mm (a-b), 1 mm (b1), 200 µm (b2). (C) Identification of hUCMSCs with CD90 and CD105. Scale bars: 200 µm (a1-a4), 50 µm (a4'). (D) Immunofluorescence staining of surface markers of hUCMSCs when co-cultured with collagen/SF scaffolds. Scale bars: 200 µm (a1-a4), 50 µm (a4''). (E) CCK-8 test of hUCMSCs when co-cultured with collagen/SF scaffolds.

Flow cytometry showed that hUCMSCs strongly expressed CD105, CD90, and CD73 to levels, and could meet the marker expression requirements of standard MSCs. After 7 days of co-culture, the cells were well adhered to the scaffold surface with many of them proliferating in the pores of the scaffold. Pseudopods extended from cells (**Figure 3A**). HE staining showed that the pores of the blank scaffolds were loose with no cell growth. In the co-cultured ones, the pores were filled with cells and the scaffolds exhibited good compatibilities with hUCMSCs (**Figure 3B**). In addition, immunofluorescence staining showed that cells adhered to the scaffolds that strongly expressed CD90 and CD105 (**Figure 3C-D**).

The CCK-8 experiment showed that OD values of the control and scaffold groups gradually increased with time. However, there were no significant differences between the two groups at each time point (*p* > 0.05). These results showed that collagen/SF scaffolds did not confer any obvious toxicity to hUCMSCs, and there was good biocompatibility between the scaffolds and hUCMSCs (**Figure 3E**).

### Complexes implantation ameliorated motor evoked potential

**Figure 4:**
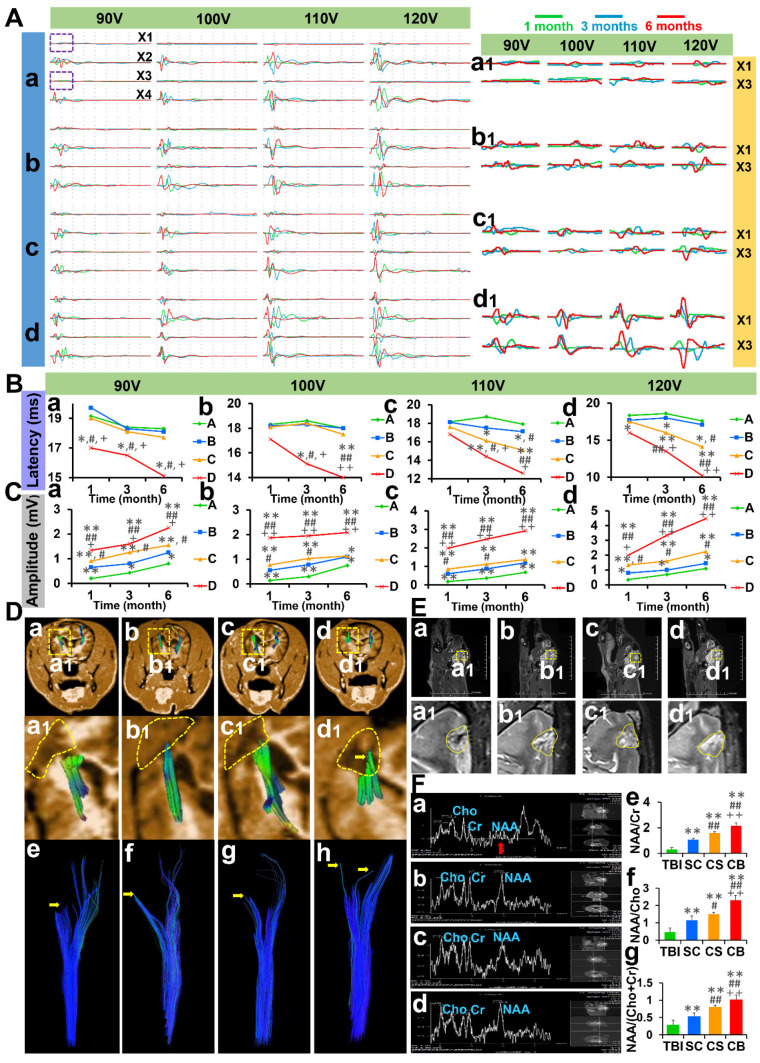
Electrophysiological examination and nuclear magnetic resonance evaluation. (A) MEP of four limbs under different levels of constant pressure stimulation (X1: left forelimb, X2: right forelimb, X3: left hindlimb, X4: right hindlimb) (B) Comparisons of MEP latency among groups under the levels of 90-120 V constant pressure stimulation (C) Comparisons of MEP amplitude among groups under the levels of 90-120 V constant pressure stimulation. (D) Observations on the integrity of corticospinal tract in TBI group, SC group, CS group, and CB group. (E) MR scanning of cerebral cortex in each group. (F) The results of spectroscopy imaging test on cerebral cortex lesions and comparisons of NAA/Cr, NAA/Cho and NAA/(Cho+Cr) values among groups. (*p < 0.05, **p < 0.01, compared with TBI group; **^#^***P* < 0.05, **^##^***p* < 0.01, compared with SC group; **^+^***P* < 0.05, **^++^***P* < 0.01, compared with CS group).

The motor evoked potential (MEP) was measured at 1, 3, and 6 months after operation (**Figure 4A**). Under 90 V constant pressure stimulation, the latency of the CB group was significantly short compared to the other groups (*p* < 0.05). While the amplitude of the CB group was significantly high compared to the other groups (TBI & SC group: *p* < 0.01; CS group: *p* < 0.05). Under 100 V constant pressure stimulation, the latency of the CB group was significantly short compared to the other groups at 3 and 6 months post-operation (3 months: *p* < 0.05; 6 months: *p* < 0.01). The amplitude of the CB group was significantly high compared to the other groups at each time point post-operation (*p* < 0.01). Under 110 V constant pressure stimulation, the latency of the CB group was significantly short than that of the TBI group (*p* < 0.01), SC group (3 months: *p* < 0.05; 6 months: *p* < 0.01) and CS group (*p* < 0.05). The amplitude of the CB group was significantly high than that of the other groups at all time points after the operation (*p* < 0.01). Under 120 V constant pressure stimulation, the latency of CB group was significantly short than that of the TBI group (1 month: *p* < 0.05; 3, 6 months: *p* < 0.01), SC group (*p* < 0.01) and CS group (3 months: *p* < 0.05; 6 months: *p* < 0.01). The amplitude of the CB group was significantly higher than that of the TBI group (*p* < 0.01), SC group (*p* < 0.01), and CS group (1 month: *p* < 0.05; 3 months, 6 months: *p* < 0.01). These results are shown in **Figure 4B-C**.

### Complexes implantation promoted the repair of cerebral cortex and corticospinal tract

Six months after the operation, the integrity of the corticospinal tract was evaluated by DTI reconstruction. There was no improvement in corticospinal tract integrity in the TBI group, SC group, and CS group after 6 months (yellow dotted frame and yellow arrow). The integrity of the corticospinal tract in the CB group was better and neonatal corticospinal tracts can be seen on the injured side (yellow dotted frame and yellow arrow) (**Figure 4D**).

Repair of the cerebral motor cortex in the TBI group was not observed. However, there was partial repair in SC and CS groups. The cerebral motor cortex in the CB group was evidently repaired (**Figure 4E**). The results of the spectroscopic imaging test on cerebral cortex lesions varied from one group to the other. The NAA/Cr values, the NAA/Cho values and the NAA/(Cho+Cr) values in the CB group were all significantly higher compared to the other three groups **(Figure 4F).**

### Complexes implantation improved limb behavior and neurological function score

**Figure 5:**
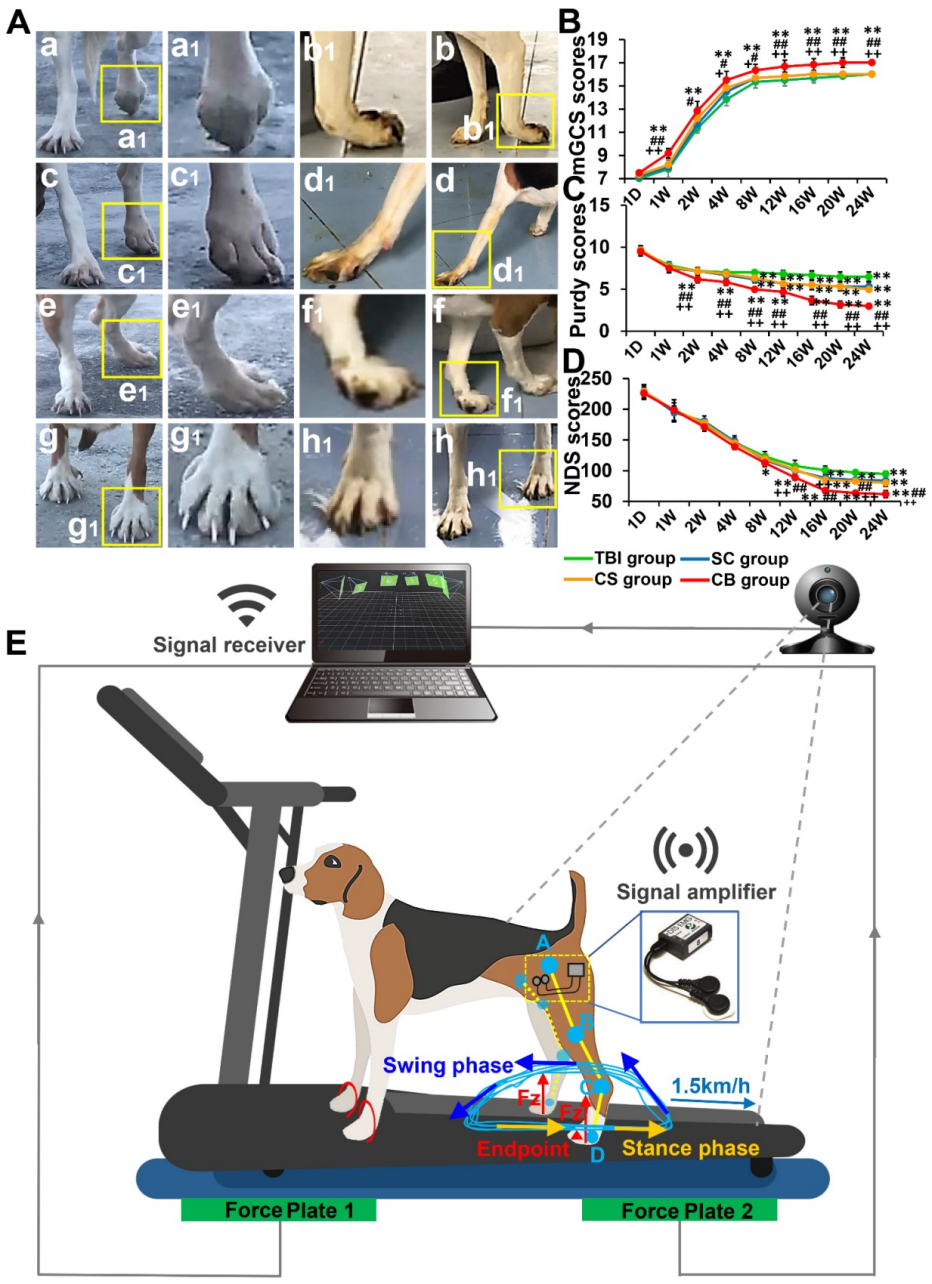
Limb behavioral evaluation and neurological function assessment. (A) The motion details of left limbs in each group. (B-D) Comparisons of mGCS, Purdy, and NDS scores among groups. (E) Schematic profile of gait detection with motion capture, sEMG and vGRF system (*p < 0.05, **p < 0.01, compared with TBI group; **^#^***p* < 0.05, **^##^***p* < 0.01, compared with SC group; **^+^***p* < 0.05, **^++^***p* < 0.01, compared with CS group).

Six months after the operation, the movement status of the limbs was assessed. In the TBI group, the left limb joints were seriously deformed, rigid, and weak, with back extension disorders. It was difficult for the left limbs to raise up, and they did not exhibit any weight-bearing capacity. The SC group and CS group exhibited a better weight-bearing ability, however, joint weakness and slippage were still obvious. In the CB group, the weight-bearing capacity of the left limbs was significantly enhanced with the left and right limbs being coordinated and symmetrical during exercise (**Figure 5A**).

Neurological functions were evaluated at 1 day, 1 week, 2 weeks, 4 weeks, 8 weeks, 12 weeks, 16 weeks, 20 weeks, and 24 weeks after the operation. On day one, there were no significant differences in neurological function scores between groups. The mGCS scores of the CB group were significantly high compared to the TBI group (*p* < 0.01), SC group (2, 4 weeks: *p* < 0.05; 1, 8, 12, 16, 20, 24 weeks: *p* < 0.01) and CS group (8 weeks: *p* < 0.05; 1, 12, 16, 20, 24 weeks: *p* < 0.01) (**Figure 5B**). The Purdy scores of the CB group were better than those of the other groups at each time point after 2 weeks (*p* < 0.01) (**Figure 5C**). The NDS scores of CB group were significantly better than those of the TBI group (8 weeks: *p* < 0.05; 12, 16, 20, 24 weeks: *p* < 0.01), SC group and CS group (12, 16, 20, 24 weeks: *p* < 0.01) (**Figure 5D**).

### Complexes implantation enhanced gait recovery in hemiplegic limbs

**Figure 6:**
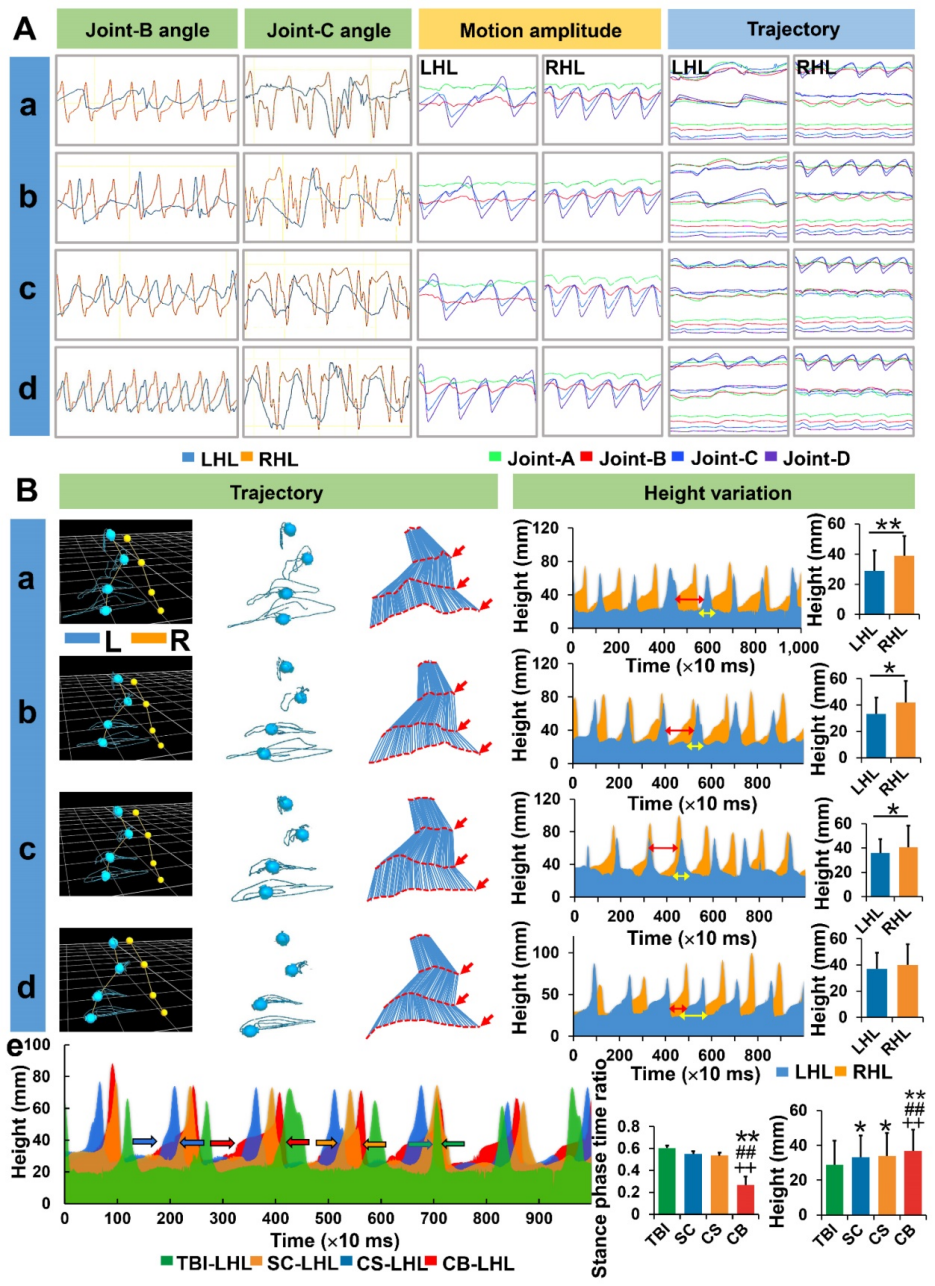
Assessment of joint motion details under motion capture system. (A) Angle changes of joint B, C along with motion amplitude and trajectories of the left hindlimbs in each group. (B) Real-time trajectory tracking and height changes of each joint for left hindlimbs in each group. (LHL: left hindlimb, RHL: right hindlimb) (*p < 0.05, **p < 0.01, compared with TBI group; **^#^***p* < 0.05, **^##^***p* < 0.01, compared with SC group; **^+^***p* < 0.05, **^++^***p* < 0.01, compared with CS group)

Six months after the operation, gait characteristics of the animals in each group were assessed using the Vicon Motion Capture System (Oxford Metrics Limited, UK). Joint B and joint C were evaluated because they exhibited the greatest changes in angle and amplitude. In the TBI group, it was difficult to initiate movements in hemiplegia limbs while joint instability, slippage or stiffness, and limb dragging were particularly severe** (Video S1)**. Irregular angle changes and asynchronism of frequency were obvious on the left limbs. Compared to the TBI group, angle changes of joint B and joint C in the SC and CS groups were regular, however, there were serious frequency dislocations on the left limbs. Gait was markedly improved in the CB group **(Video S1)**, and the best regularity of angle change and frequency synchronization can be seen on the left hindlimbs. The same phenomenon occurred with regards to motion amplitude and trajectory variation of each joint. Motion amplitude and trajectory of hemiplegic limb joints were both observed the closest to the healthy limbs in the CB group **(Figure 6A)**.

In the TBI group, the trajectories of the four joints of the left hindlimb were disordered and irregular. Movements between joints were uncoordinated, the time of limb dragging (stance-phase) on the ground was prolonged while the swing-phase time was significantly shortened. The lifting height of the left hindlimb from the endpoint was significantly lower than that of the right hindlimb (*p* < 0.01). The trajectories of the four joints in SC and CS groups were improved, and were more regular and better coordinated than in the TBI group. The swing-phase time proportion in the CS group was slightly prolonged compared to the TBI group (*p* < 0.05, CS group *vs* TBI group). However, the height of the lifted left hindlimb was still significantly lower than that of the right hindlimb (*p* < 0.05). In the CB group, the trajectories of the four joints of the left hindlimbs were improved and were more regular and coherent. The swing phase time proportion was prolonged while the stance-phase time was significantly short compared to the other three groups (*p* < 0.01). There was no significant difference in lift height between the left and right hindlimbs (*p* > 0.05). Compared to the other groups, the trajectory of the left hindlimb height change was more regular and closer to the right limb, and the absolute height of the left hindlimb was significantly higher than in the other groups (*p* < 0.01)** (Figure 6B)**.

### Complexes implantation improved myodynamia and electromyography of hemiplegic limbs

**Figure 7:**
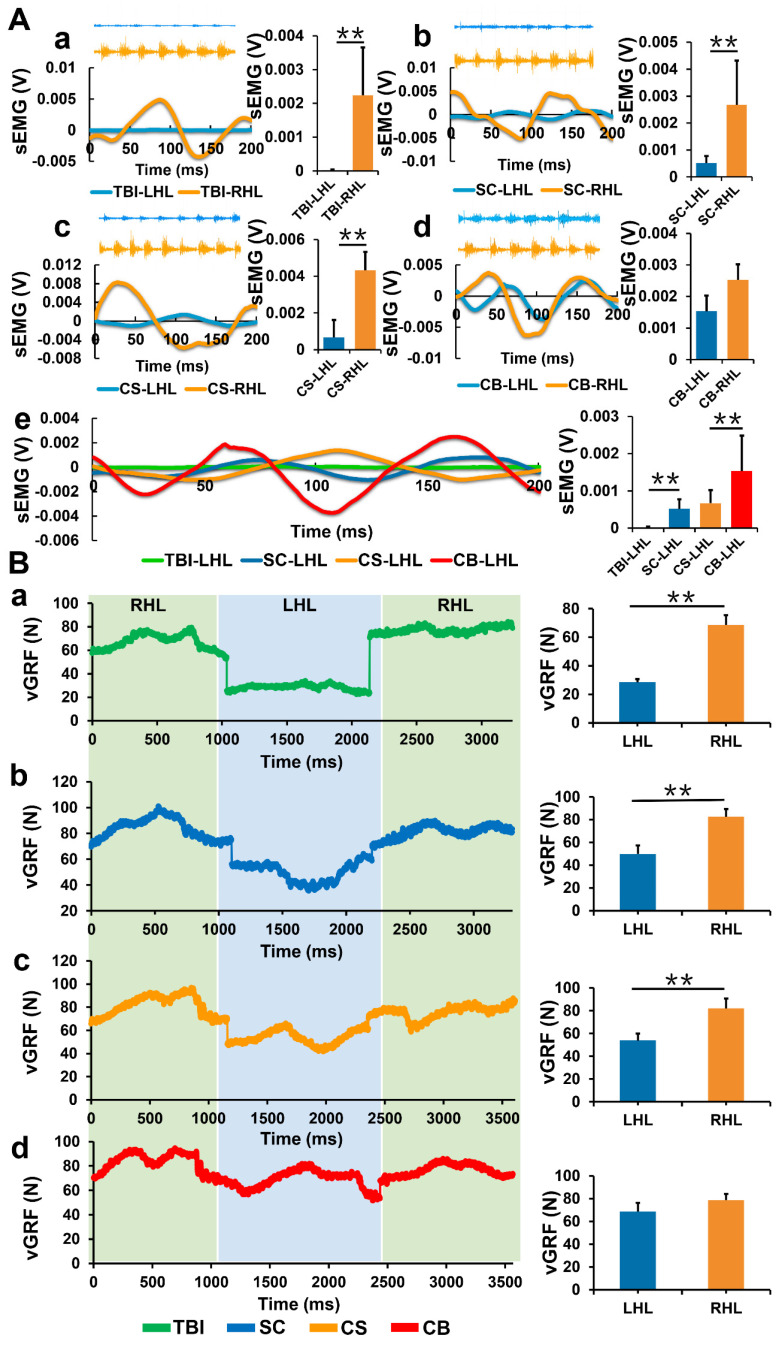
Detection of sEMG and vGRF. (A) Real-time sEMG changes of left and right hindlimbs in each group. (B) Changes in vGRF values of left and right hindlimbs during a single kinematic cycle in each group. (LHL: left hindlimb, RHL: right hindlimb) (**p< 0.01).

While detecting the sEMG activity of limbs, joint stiffness and abnormal muscle activity were frequent during exercise in the TBI group. The sEMG of the left hindlimbs was significantly weak compared to that of the right hindlimbs (*p* < 0.01). Compared to the TBI group, the sEMG of the left hindlimbs in the SC and CS groups improved, but were still significantly weak compared to the right hindlimbs (*p* < 0.01). The sEMG of the left hindlimbs in the CB group was significantly improved, however, there was no significant difference between the left and right hindlimbs (*p*>0.05). The absolute value of sEMG in the SC group was significantly stronger than that in the TBI group (*p* < 0.01). sEMG in the CB group was significantly stronger compared to the other groups (*p* < 0.01) (**Figure 7A**).

In the TBI, SC and CS groups, the vGRF values of the left hindlimbs were significantly low compared to the right hindlimbs (*p* < 0.01). There was no significant difference in vGRF between the left and right hindlimbs in the CB group **(Figure 7B)**. vGRF represents the vertical reaction force of the ground to the limbs, the value is equal to the force exerted on the ground by the limbs when exercising. vGRF can indirectly reflect the muscular strength of the limbs. The results suggested that the muscular strength of the hemiplegic limbs in the CB group was significantly improved compared to the other groups.

### Complexes implantation stimulated brain injury repair and inhibited glial proliferation

**Figure 8:**
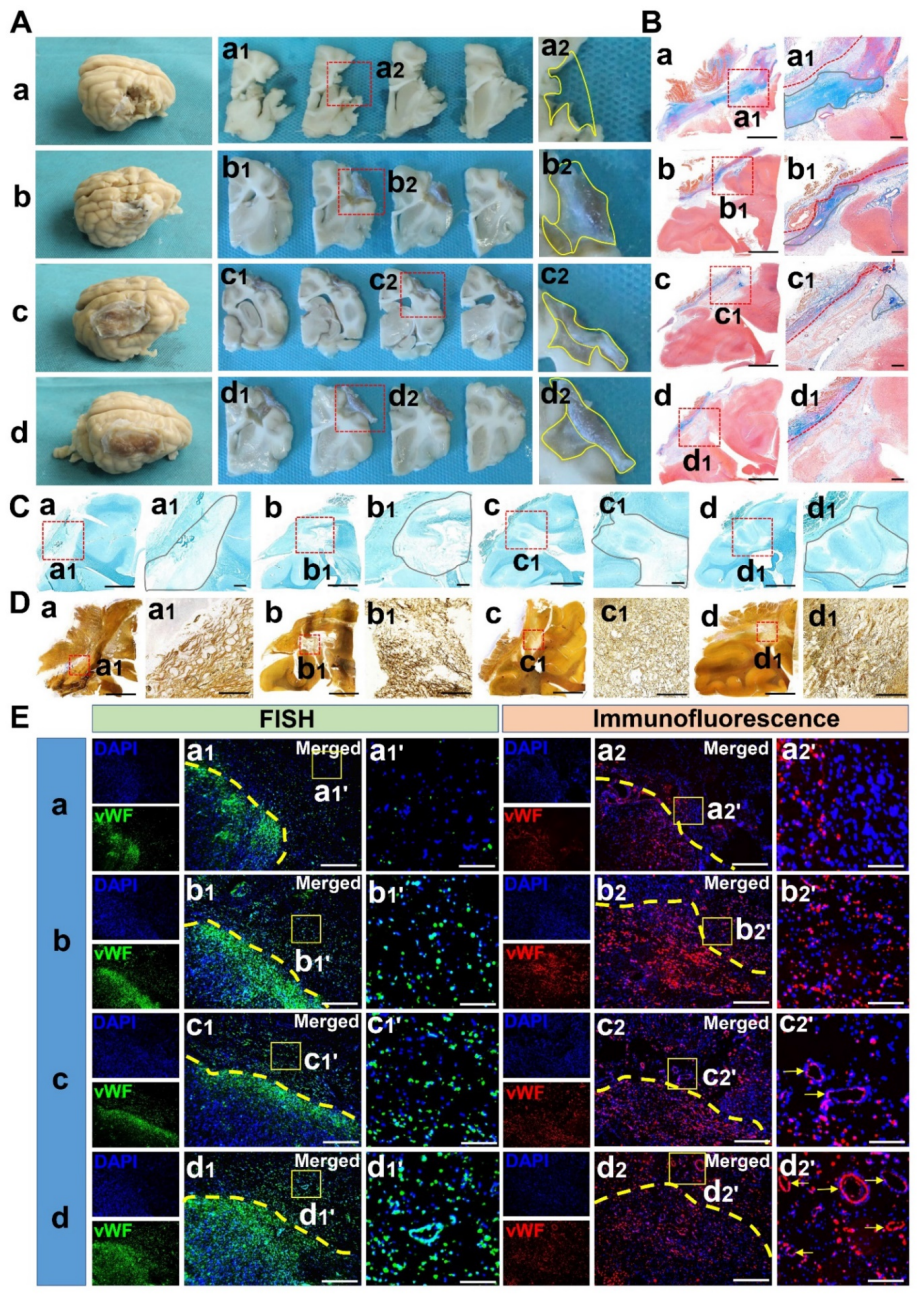
Gross and staining observation of cerebral cortex repair along with vascular regeneration. (A) General view of lesion filling and cerebral cortex repair in each group. (B) Glial hyperplasia detection of cerebral cortex lesion by Masson staining in each group. Scale bars: 1 cm (a-d), 0.2 cm (a1-d1) (C) LFB staining of cerebral cortex lesion in each group. Scale bars: 1 cm (a-d), 0.2 cm (a1-d1). (D) Silver staining of cerebral cortex lesion in each group. Scale bars: 1 cm (a-d), 100 µm (a1-d1). (E) mRNA and protein expression of vWF in the cerebral cortex lesion of each group. Scale bars: 200 µm (a1-d1 & a2-d2), 50 µm (a1'-d1' & a2'-d2').

Serious structural deficiency was observed in the TBI group. In the SC and CS groups, the lesion was partially filled, and most of the neonatal tissue was made up of thickened meninges and hypertrophic glial scars. In the CB group, the lesions were filled without thickened meninges and hypertrophic glial scars (**Figure 8A**). Masson staining showed that there were many glial fibers in the lesions of the TBI group, while the glial fibers in SC and CS groups were significantly reduced. In the CB group, the lesions were well repaired without the formation of glial fibers (**Figure 8B**). LFB staining showed that there was no blue myelin sheath in the lesions of the cerebral cortex in the TBI group. In the SC and CS groups, the local structure of lesions was disordered, although a few regenerated myelin sheaths appeared. In the CB group, numerous blue-stained myelin sheaths were observed (**Figure 8C**). Silver staining showed that there was no regeneration of nerve fibers and synapses in the TBI group, while vacuolar necrosis-like structures were abundant. Although a few regenerative nerve fibers were observed in the SC and CS group, structural disorganization was still remarkable. In the CB group, the lesions were well repaired with many regenerative nerve fibers and synapse-like structures (**Figure 8D**).

### Complexes implantation promoted vascular regeneration

Immunofluorescence staining showed that more positive cells expressing vWF at the mRNA and protein levels could be observed in SC and CS groups compared to the TBI group (*p* < 0.01). There were more positive cells in the CB group compared to the other groups (*p* < 0.01) (**Figure 8E**) **(Figure S6A-B)**. This implied that angiogenesis was weak in the TBI group, and there was no blood supply reconstruction. In the CB group, there were a lot of new blood vessels, and the blood supply reconstruction was significantly more obvious compared to the other groups.

### Complexes implantation induced the regeneration of neuron cytoskeleton, axon and enhanced the formation of synapse and myelination

**Figure 9:**
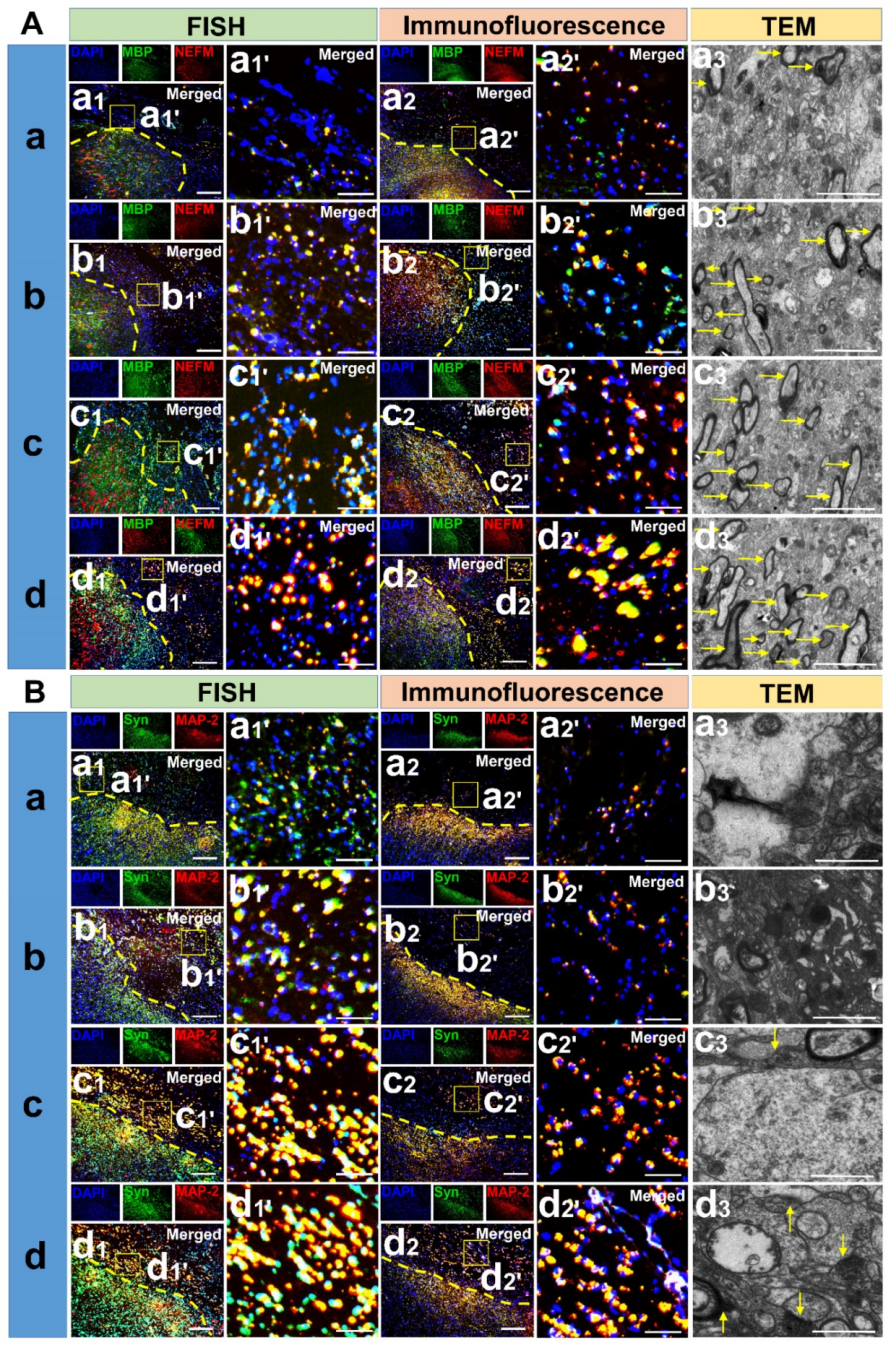
Detection of neural regenerative markers among groups. (A) mRNA and protein expression of MBP and NEFM along with TEM scanning in the cerebral cortex lesion of each group. Scale bars: 200 µm (a1-d1 & a2-d2), 50 µm (a1'-d1' & a2'-d2'), 5 µm (a3-d3). (B) mRNA and protein expression of Syn and MAP-2 along with TEM scanning in the cerebral cortex lesion of each group. Scale bars: 200 µm (a1-d1 & a2-d2), 50 µm (a1'-d1' & a2'-d2'), 5 µm (a3-d3).

From the in-situ hybridization and immunofluorescence staining results, more positive cells co-expressing MBP and NEFM at the mRNA and protein levels could be observed in SC and CS groups than in the TBI group (*p* < 0.01). There were more positive cells in the CB group compared to the other groups (*p* < 0.01) (**Figure 9A**) (**Figure S6C-D**). These results implied that axonal regeneration and demyelination was most obvious in the CB group. Meanwhile, more positive cells co-expressing Syn and MAP-2 at the mRNA and protein level could be observed in the SC and CS groups than in the TBI group (*p* < 0.01). There were more positive cells in the CB group than in the other groups (CB *vs* CS: *p* < 0.05; CB *vs* TBI & SC: *p* < 0.01), (**Figure 9B**) (**Figure S6E-F**). These results indicated that cytoskeleton and axon regeneration was very low in the TBI group while it was highest in the CB group. In addition, new myelin sheaths and synaptic structures in the TBI group were very few. Even though the number of new myelin sheaths in the SC and CS groups was higher compared to the TBI group, synaptic structure formation was not detected. Re-myelination and synaptic structure regeneration were the most notable in the CB group (**Figure 9A-B**).

### Complexes enhanced hUCMSCs survival and the formation of neuronal tissue

Apart from the specific surface markers of hUCMSCs in the CB group **(Figure S3)**, there were more DCX^+^ cells in the CB group compared to the SC group (*p* < 0.01) (**Figure S4**) (F**igure S6G**). The number of NeuN^+^ cells in the CB group was significantly high compared to the SC group (*p* < 0.01) (**Figure S5**) (**Figure S6H**).

### Complexes implantation regulated systemic inflammatory factor levels in the acute and chronic stages of traumatic brain injury

In the acute stage of TBI, the expression of IL-6 in the TBI group was elevated compared to the other groups (*p* < 0.01), while the level of IL-6 in the CB group was lower compared to the the CS group (*p* < 0.01) **(Figure S6I)**. The expression level of TNF-α in the TBI group was significantly high compared to the CS and CB groups (*p* < 0.01), while its expression level in the CS group was significantly high compared to the CB group (*p* < 0.05) **(Figure S6J)**. The expression of IL-10 in the TBI group was significantly suppressed compared to the SC group (*p* < 0.01), CS group (*p* < 0.05) and CB group (*p* < 0.01), while its expression level was significantly elevated in the CB group compared to the CS group (*p* < 0.01) (**Figure S6K**). The ratio of IL-10 to IL-6 in SC, CS, and CB groups was significantly high than that in the TBI group (*p* < 0.01), while the ratio in the CB group was significantly high than that in the SC and CS groups (*p* < 0.01) (**Figure S6L**). In the chronic stages of TBI, the expression levels of IL-6 in the TBI group were significantly high compared to the other groups (*p* < 0.01). The level of IL-6 in the CB group was significantly low compared to the SC group (*p* < 0.05) and the CS group (*p* < 0.01) (**Figure S6M**). Meanwhile, the expression levels of TNF-α in the TBI group was significantly high than that in the CS group (*p* < 0.05) and CB group (*p* < 0.01) (**Figure S6N**). The expression level of IL-10 in the TBI group was significantly low compared to the SC group (*p* < 0.01), CS group (*p* < 0.05), and CB group (*p* < 0.01) (**Figure S6O**). The ratio of IL-10 to IL-6 in the CB group was significantly low than that in the other groups (*p* < 0.01) (**Figure S6P**).

## Discussion

The global annual incidences of TBI occur in more than 50 million people 21. The burden caused by TBI is profound for families and social societies. The damage inflicted to key functional areas of the brain is the main cause of long-term disability. Advances in tissue engineering have provided avenues for the repair of neural trauma and rehabilitation of motor functions 22. The implantation of biological scaffolds with seed cells has exhibited broad clinical applications. Studies are devoted to exploring suitable materials for neural regeneration. It has been found that collagen and silk fibroin are two favorable options in the repair of spinal cord injury (SCI) on the basis of our previous studies. To determine the applicability of these materials in TBI repair, we fabricated a scaffold with collagen and silk fibroin and implanted them with hUCMSCs in the brain trauma foci. It was shown that some representative markers of neurogenesis were significantly expressed while the gait of the animals were notably improved. Therefore, co-implantation of collagen/silk fibroin scaffold and hUCMSCs promoted the reconstruction of neural networks and enhanced the recovery of motor functions.

Safety and biocompatibility are two critical factors for material selection in neural regeneration studies. Studies have shown that biomaterials can fill the excessive lesions, determine the fate of seed cells 6, improve local microenvironment, inhibit scar proliferation and promote axonal regeneration 23, 24. However, the development of biological scaffolds for TBI repair is limited because the structure and functions of the brain are much more complex when compared to the spinal cord. Various injury mechanisms also inhibit TBI repair 25. Collagen is a matrix component that is widely distributed extracellularly. It does not exhibit any immunogenicity after pretreatment and purification. Favorable degradation rates and biocompatibility also make it an excellent choice for many biological studies 26-28. However, certain defects such as poor mechanical properties and weak thermal stability impose some limits in its use 29, 30. Surprisingly, as a material with low immunogenicity, SF has gratifying mechanical properties and thermal stability 31-33. To retain the advantages and make up for the defects of materials, we crosslinked the collagen and SF to form a complex**,** instead of making a liquid or semi-liquid form 34, 35. We fabricated a porous structure of the complex, which avoided the collapse of the scaffold and provided sufficient space for the survival, growth as well as differentiation of seed cells. In addition, it forms a channel for nutrient and metabolic waste exchange and newborn axons crawling. The chemical bonds of scaffolds also facilitate hUCMSCs adherence 36.

As multi-potential stem cells, MSCs differentiate into neuronal structures under certain circumstances. However, the local microenvironment after TBI suppresses the survival and differentiation of MSCs and may induce their differentiation into glial cells 37, 38. Fortunately, the outcome of Masson staining indicated that there was no glial hyperplasia in the trauma foci after the co-implantation of scaffolds and hUCMSCs. While significant formation of glial scar was observed in SC group, fate tracking is of great significance in cell transplantation related therapy 39. In this study, we arranged the in vitro tracing of seed cells. The co-expression of CD-90 and CD-105 confirmed the long-term survival of hUCMSCs. Moreover, as representative markers of newborn neurons (DCX) and mature neurons (NeuN), the immunohistochemical results confirmed that hUCMSCs implantation enhanced regeneration of neuronal tissue under the shelter of scaffolds in CB group 40. Indicating that collagen/silk fibroin scaffolds may create a favorable microenvironment for the survival and oriented differentiation of seed cells.

The re-networking of blood vessels and motor pathway is the premise of functional improvement. Although the MRI results showed that lesions of several groups were filled, the specific composition and functions of the filled area have not been established. Therefore, a spectroscopic imaging test was performed to analyze metabolitic compositions such as neurometabolites, amino acids, choline, and other substances. In the TBI group, the NAA/Cr, NAA/Cho, NAA/(Cr+Cho) ratios were significantly low compared to the other groups. Studies have shown that a decline of the above ratios is closely associated with the severity of brain trauma 41, 42. In the ISH and immunofluorescence staining, vWF is used as an indicator of vascular regeneration, the positive expressions of NEFM, MAP2, Syn, and MBP in both gene and protein levels intensively indicated that co-implantation of the scaffold and hUCMSCs boosted axonal regeneration, myelination and synaptic formation. In parallel to the results of ISH and immunofluorescence staining, myelination and synaptic formation were observed directly under TEM, which also corroborated the enhanced regeneration of neuronal tissue after complexes implantation. The unity of structural repair and functional recovery is a reliable standard for neurological rehabilitation. MEP is closely associated with the integrity of the corticospinal tract pathway. The significantly shortened latency and increased amplitude in the CB group demonstrated that the reconstruction of neural networks enhanced the restoration of electrical activity 43.

The TBI model used in this study is one of the highlights in this experiment. Compared to small animals, this model is closer to humans in anatomical structure, physiological functions, and pathological changes. Sham group is vital for establishing a consistent and reliable TBI across all groups to test the stem cells or collagen/silk fibroin complexes. In the present study, we didn't set it because we have evaluated the effects of craniotomy and dura incision (sham operation) on neurobehavior and motor function in TBI modeling stage 16, and we found small area craniotomy and dura incision have no adverse effects on canines in terms of neural network and motor function (**Figure S1 & Figure S2**). In the assessment of motor functions, three scoring scales were used to evaluate the level of consciousness, sensation, reflex, behavior, and limb movements. In the chronic TBI phase, the scores of the CB group were significantly high compared to those of the other groups. This implies that the implanted complexes enhanced neurological functions.

Since the scoring scales have been widely used in a large number of studies, especially neurological scoring is an indispensable part in the study of TBI and SCI. However, some possible bias may be caused in the process of scoring due to the subjective tendency and preference, thus, an objective and quantifiable evaluation system seems to be essential. Motion capture is a key technique that is important in evaluating motion state and ability in terms of kinematics and mechanics. The joint is an important pivot in limb movement. The stability of the joint plays a decisive role in the formation of integrity and healthy gait 44, 45. Motion capture depicts the motion trajectory of each joint in real-time, flexion and extension angle, swing amplitude, and a lifting height of joints to objectively and accurately reflect the individual's motion details. In the four joints of our animal model, the two most important joints for driving motions were joints B and C. The swing amplitude of these two joints was the largest and was equivalent to the ankle and knee joints of human. Injury to the right side of the brain leads to hemiplegia of the left limbs. Even though studies have established that hindlimbs play a more important role in driving motion 46-48. Adaptability training is still essential before motion capture. Under these circumstances, the hindlimbs will not be affected after a long period time of training even when the forelimbs are fixed 49, 50. The results of the motion capture showed that the angle changes and trajectory were more regular in CB group. Moreover, the results of height changes and stance phase time ratio also demonstrated that canines in CB group can lift up limbs instead of dragging limbs on the ground in other groups. Compared to the traditional technique of needle EMG detection, sEMG has the advantage of wireless transmissions without loss of accuracy 51. Coordinating with the results of DTI and MEP detection, great improvement of sEMG in CB group established the integrity of the superior neural circuit. vGRF can reflect muscle strength and bearing capacity of the limbs, when hemiplegia occurs in one limb, the muscle strength and the bearing capacity decreases, the vertical force exerted on the ground during exercise is limited and the strength to maintain balance falls heavily on the healthy side of the limbs. Therefore, this technique reflects the situation of hemiplegia and rehabilitation through quantitative comparisons.

Inflammation affects neural rehabilitation 52. The levels of IL-10 and IL-6 and their ratios reflect the balance of inflammation and anti-inflammation. Implantation of the complexes regulated systemic inflammation levels in the acute and chronic TBI stages. This effect could be attributed to the robust anti-inflammatory effects of hUCMSCs in neurological diseases such as stroke, epilepsy, or TBI 53.

Despite of great potential for clinical transformation, issues still remain to be addressed. TBI may result in long-term motor deficits in adult mammals as its poor microenvironment limits neural regeneration. The survival and oriented differentiation of seed cells are global challenges. Biomaterials can be improved through physical, chemical, or gene modifications 54. The mechanisms of collagen/SF scaffolds in repairing CNS trauma and the optimal time window of implantation have not yet been established 55.

## Conclusion

The porous collagen/SF scaffolds have good physical properties and favorable biocompatibilities with hUCMSCs. Implantation of complexes developed by the co-culture of collagen/SF scaffolds and hUCMSCs not only repairs the anatomical structure of traumatic lesions, but also restores the gait of hemiplegic limbs after TBI.

## Supplementary Material

Supplementary figures.

Supplementary video.

## Abbreviations

TBI: traumatic brain injury
MSCs: mesenchymal stem cells
SF: silk fibroin
MEP: motor evoked potential
MRI: magnetic resonance imaging
DTI: diffusion tensor imaging
sEMG: surface electromyography
vGRF: vertical ground reaction force
ISH: in situ hybridization
LFB: Luxol Fast Blue
DCX: Doublecortin
vWF: von Willebrand factor
MBP: myelin basic protein
MAP-2: microtubule-associated protein-2
NEFM: neurofilament M
Syn: synapsin
SEM: scanning electron microscopy
mGCS: modified Glasgow Coma Scale
