# Supplementary material for: Implantation of regenerative complexes in traumatic brain injury canine models enhances the reconstruction of neural networks and motor function recovery
Source: Theranostics. 2021 Jan 1;11(2):768–88. doi: 10.7150/thno.50540 (PMC7738861; doi:10.7150/thno.50540)
Supplement: Supplementary file 1 — Supplementary figures. [file thnov11p0768s1.pdf]

Supplementary materials

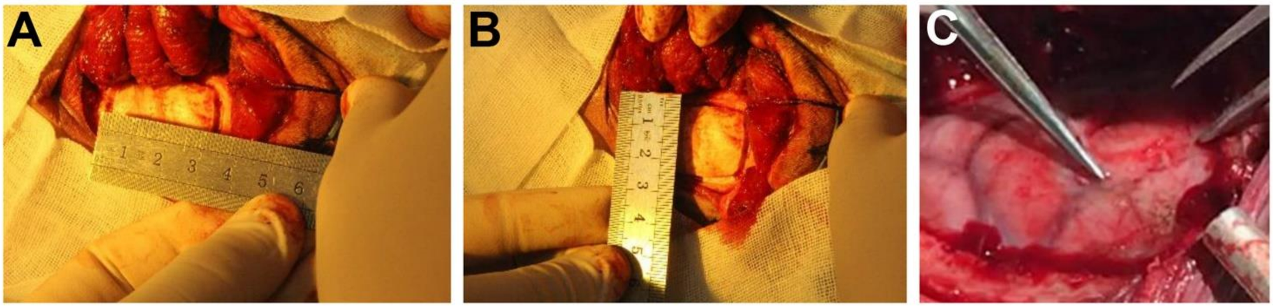

Supplementary figure 1. Sham operation (craniotomy and dura incision) was performed in sham group. (A-B) The skull defect was designed small and the skull defect located under the temporal muscle and occipital muscle. (C) Dura incision was performed after craniotomy.

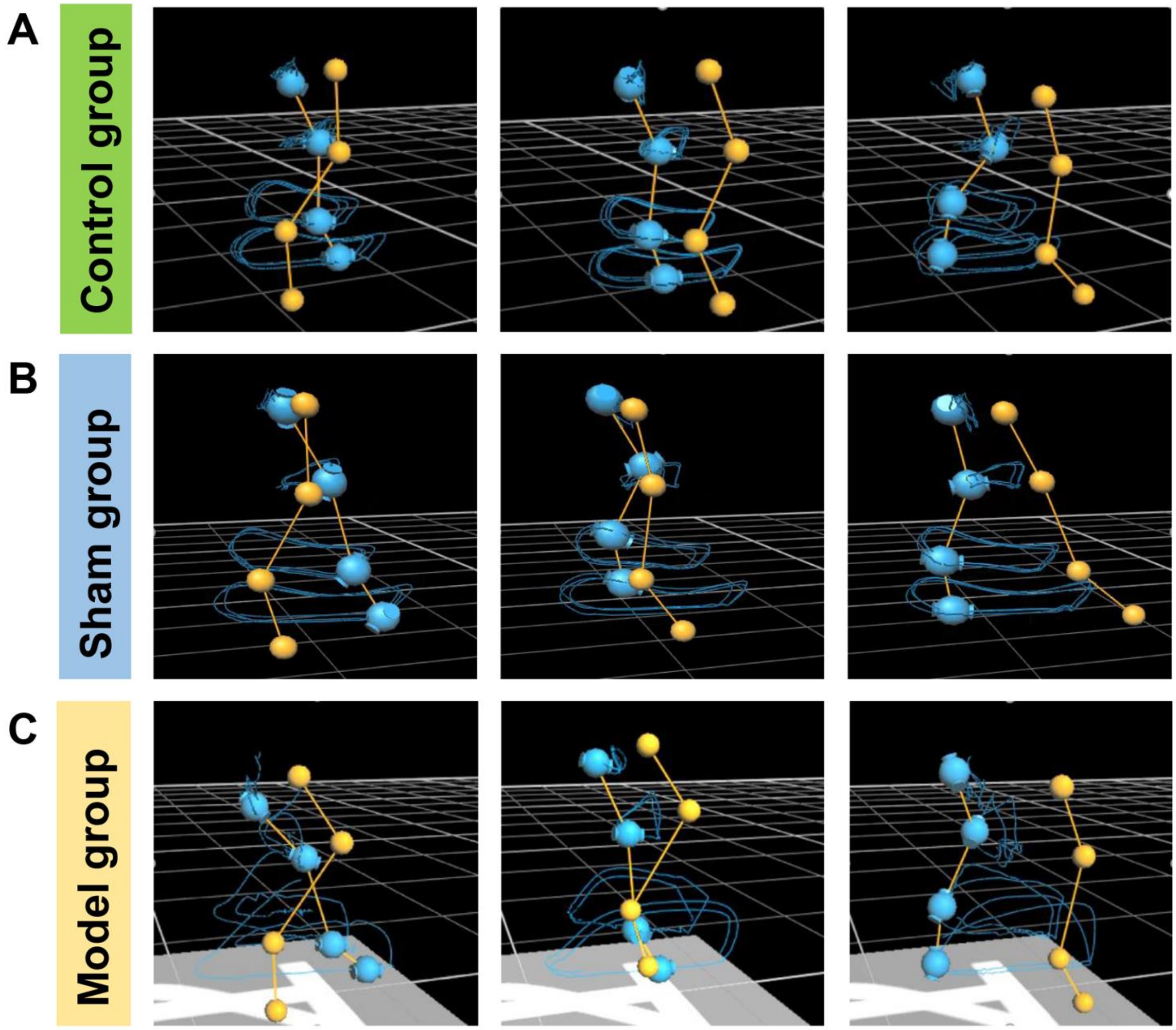

Supplementary figure 2. Comparisons of gait characteristics among groups. There is no significant difference between sham group and control group (A-B), trajectories of joints were regular in these two groups while those of them in TBI group were obviously irregular (C).

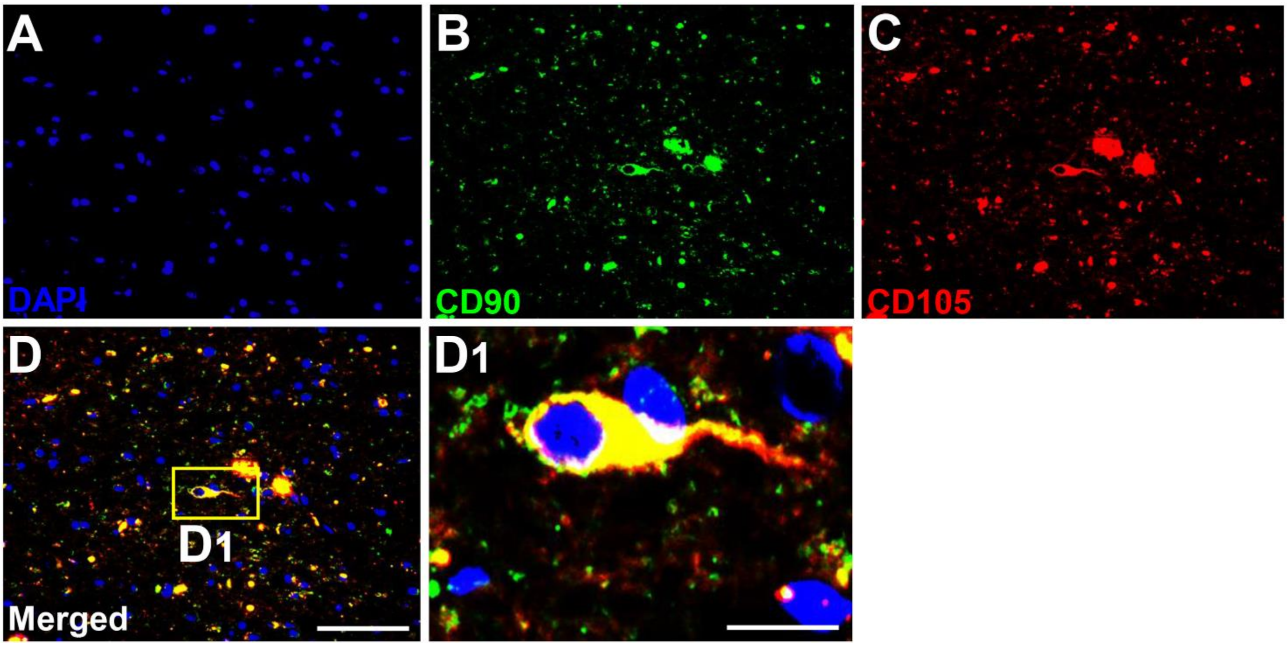

Supplementary figure 3. Stem cell fate tracing with CD90 and CD105 staining. Scale bars: 200 μm (D), 50 μm (D1).

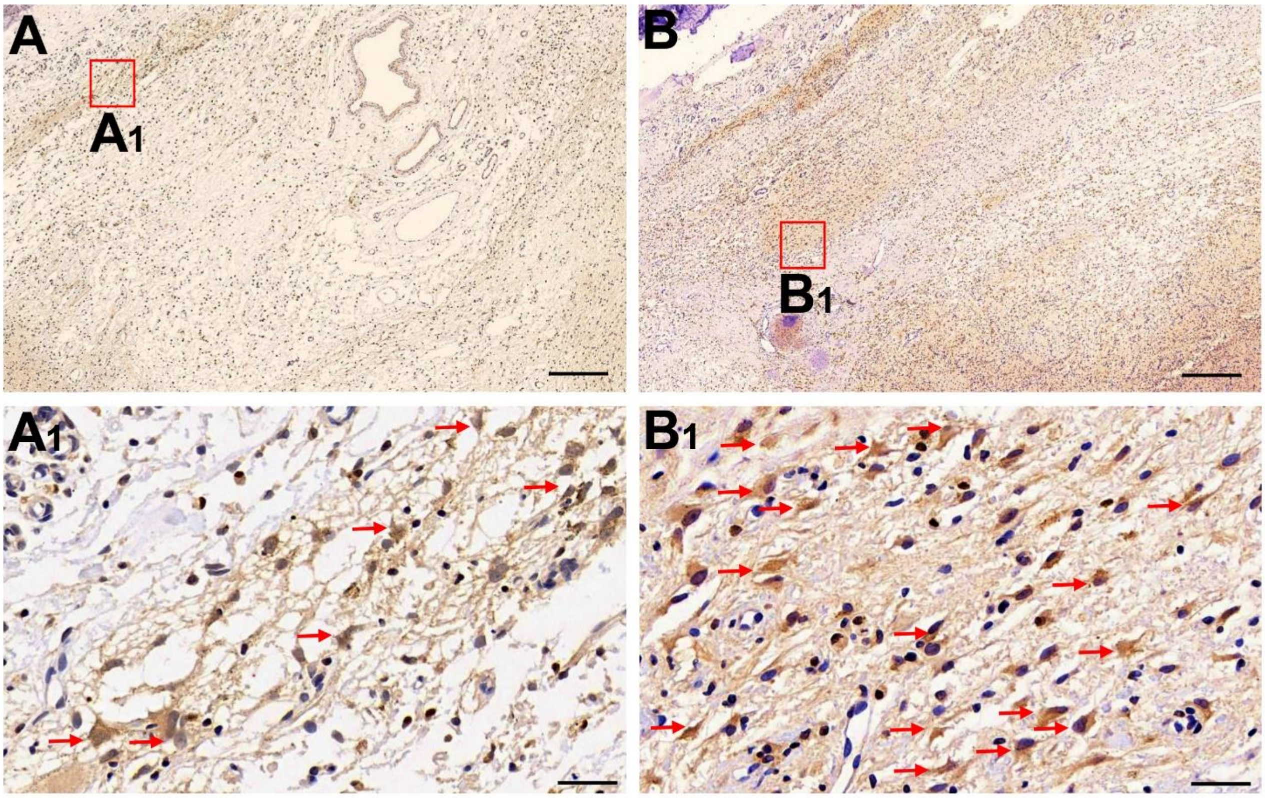

Supplementary figure 4. Stem cell fate tracing with DCX staining. DCX staining of traumatic foci in SC group (A-A1) and CB group (B-B1). Scale bars: 100 μm (A-B), 10 μm (A1-B1).

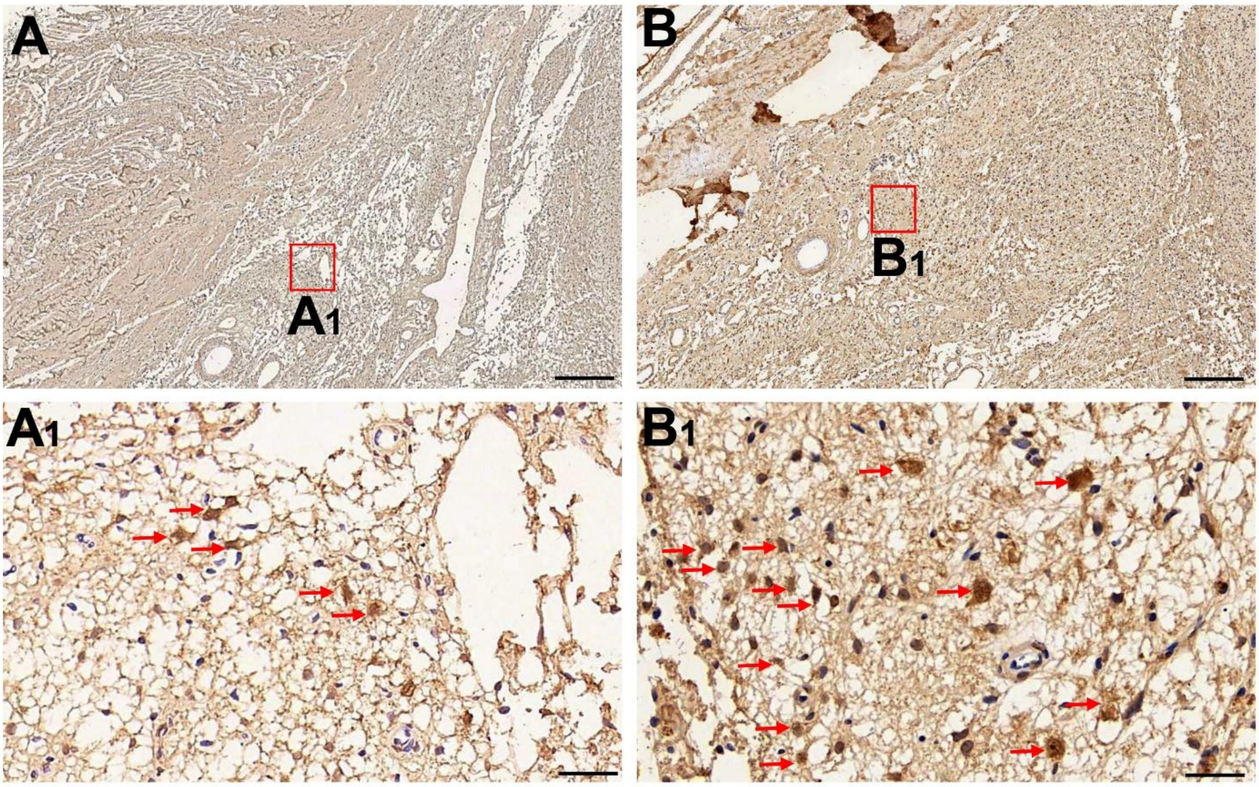

Supplementary figure 5. Stem cell fate tracing with NeuN staining. NeuN staining of traumatic foci in SC group (A-A1) and CB group (B-B1). Scale bars: 100 µm (A-B), 10 µm (A1-B1).

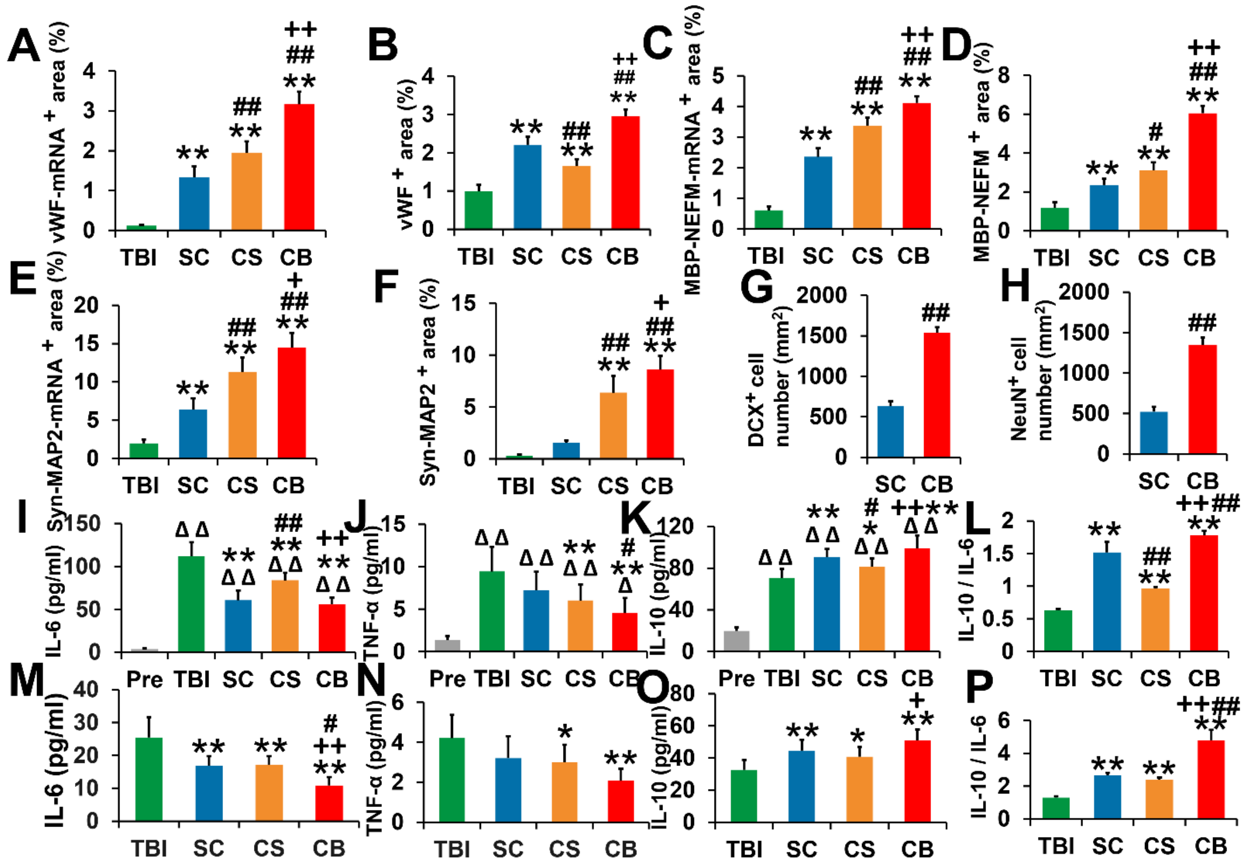

Supplementary Figure 6: Statistical analysis of immunofluorescence staining experiment and systemic inflammatory reaction detection. Comparisons of positive cells of vWF (A-B), MBP-NEFM (C-D) and Syn-MAP2 (E-F) expression at the level of mRNA and protein among groups. Comparisons of DCX^+ (G) and $NeuN^+$ (H) cells between SC and CB group. Comparisons of IL-6 (I), TNF-α (J), and IL-10 (K) level among groups before and 1 week after operation. (L) Comparisons of IL-10/IL-6 value among groups 1 week after operation. Comparisons of IL-6 (M), TNF-α (N), and IL-10 (O) level among groups 6 mon after operation. (P) Comparisons of IL-10/IL-6 value among groups 6 mon after operation. ($^*p < 0.05$, $^{**}p < 0.01$, compared with TBI group; $^{\#}p < 0.05$, $^{\#\#}p < 0.01$, compared with SC group; $^{+}p < 0.05$, $^{++}p < 0.01$, compared with CS group)

Supplementary video 1: Comparisons of gait characteristics among groups under motion capture system. (A) Back view of gait details in each group (B) Left side view of gait details in each group (C) Right side view of gait details in each group.
